# Hepatic loss of *Lissencephaly 1* (*Lis1*) induces fatty liver and accelerates liver tumorigenesis in mice

**DOI:** 10.1074/jbc.RA117.001474

**Published:** 2018-02-23

**Authors:** Xiaoling Li, Liansheng Liu, Ran Li, Ailing Wu, Jinqiu Lu, Qingzhe Wu, Junling Jia, Mujun Zhao, Hai Song

**Affiliations:** From the ‡ Life Sciences Institute and Innovation Center for Cell Signaling Network, Zhejiang University, Hangzhou 310058, China and; the § State Key Laboratory of Molecular Biology, Institute of Biochemistry and Cell Biology, Shanghai Institutes for Biological Sciences, Chinese Academy of Sciences, Shanghai 200031, China

**Keywords:** endoplasmic reticulum stress (ER stress), Golgi, liver metabolism, liver cancer, hepatocyte, mouse genetics, Lis1, nonalcoholic fatty liver disease, ploidy, steatosis

## Abstract

The liver is a major organ in lipid metabolism, and its malfunction leads to various diseases. Nonalcoholic fatty liver disease, the most common chronic liver disorder in developed countries, is characterized by the abnormal retention of excess lipid within hepatocytes and predisposes individuals to liver cancer. We previously reported that the levels of Lissencephaly 1 (LIS1, also known as PAFAH1B1) are down-regulated in human hepatocellular carcinoma. Following up on this observation, we found that genetic deletion of *Lis1* in the mouse liver increases lipid accumulation and inflammation in this organ. Further analysis revealed that loss of *Lis1* triggers endoplasmic reticulum (ER) stress and reduces triglyceride secretion. Attenuation of ER stress by addition of tauroursodeoxycholic acid (TUDCA) diminished lipid accumulation in the *Lis1*-deficient hepatocytes. Moreover, the Golgi stacks were disorganized in *Lis1*-deficient liver cells. Of note, the *Lis1* liver-knockout mice exhibited increased hepatocyte ploidy and accelerated development of liver cancer after exposure to the liver carcinogen diethylnitrosamine (DEN). Taken together, these findings suggest that reduced *Lis1* levels can spur the development of liver diseases from steatosis to liver cancer and provide a useful model for delineating the molecular pathways that lead to these diseases.

## Introduction

Liver regulates several key aspects of lipid metabolism including fatty acid β-oxidation, lipogenesis, and lipoprotein uptake and secretion in response to nutritional and hormonal signals (1). Malfunctioning of these processes can lead to various diseases including fatty liver disease and liver cancer. Nonalcoholic fatty liver disease (NAFLD)^2^ is characterized as an abnormal retention of excess lipid within hepatocytes (steatosis). NAFLD affects one-third of adults and an increasing number of children in developed countries and is strongly associated with obesity and insulin resistance (2, 3). Hepatic steatosis can proceed to nonalcoholic steatohepatitis (NASH), a condition associated with hepatocyte injury, inflammation, and fibrosis. NASH can further progress to cirrhosis and liver cancer (4, 5). Prolonged endoplasmic reticulum (ER) dysfunction is implicated in the regulation of liver lipid metabolism as well as in the development of hepatic steatosis and NASH (6–8). Mitochondrial dysfunction is also involved in the onset and/or progression of NASH (9). Although significant progress has been made, the pathogenesis of NAFLD remains poorly understood.

*LIS1* was first identified to be responsible for type I lissencephaly, a severe neuronal developmental disease (10). Genetic analysis of patients with type I lissencephaly has revealed mutations or hemizygous deletions in the *LIS1* gene (11). *Lis1* knockout mice further demonstrated a dosage-sensitive neuronal-specific role for *Lis1* in neuronal migration throughout the brain (12, 13). LIS1 was previously shown to be involved in the dynein motor complex that determines microtubule-associated movement of organelles and membrane vesicles, and maintenance of the integrity of the Golgi apparatus and mitotic spindle assembly (14–18). In addition to mediating dynein function, LIS1 also regulates actin dynamics and Rho GTPase signaling (19–21). Dynein complex was implicated to the process of the transport of newly synthesized membrane material from the ER to Golgi apparatus (22), thus supporting a possible mechanistic link LIS1 and secreted protein trafficking. Indeed, one study indicated that LIS1 is involved in the vesicular trafficking between the ER and Golgi (23). In addition to brain development, the functions of *Lis1* during organgenesis have been investigated, such as skin, the organ of Corti, and the hematopoietic system (24–27). However, the role of *Lis1* in liver development and homeostasis remains elusive.

Our previous study showed that the mRNA and protein levels of LIS1 are down-regulated in about 70% of hepatocellular carcinoma tissues, and this down-regulation was significantly associated with tumor progression (28). Functional studies showed that knockdown *Lis1* expression results in cellular transformation in NIH3T3 (28). To further elucidate the role of *Lis1* in liver homeostasis and disease, we generated hepatocyte-specific *Lis1* knockout mice. We showed that loss of *Lis1* leads to a progressive development of liver disease from hepatic steatosis, NASH to liver cancer.

## Results

### Selective inactivation of Lis1 in the liver leads to accumulation of lipids in hepatocytes

**Figure 1.**
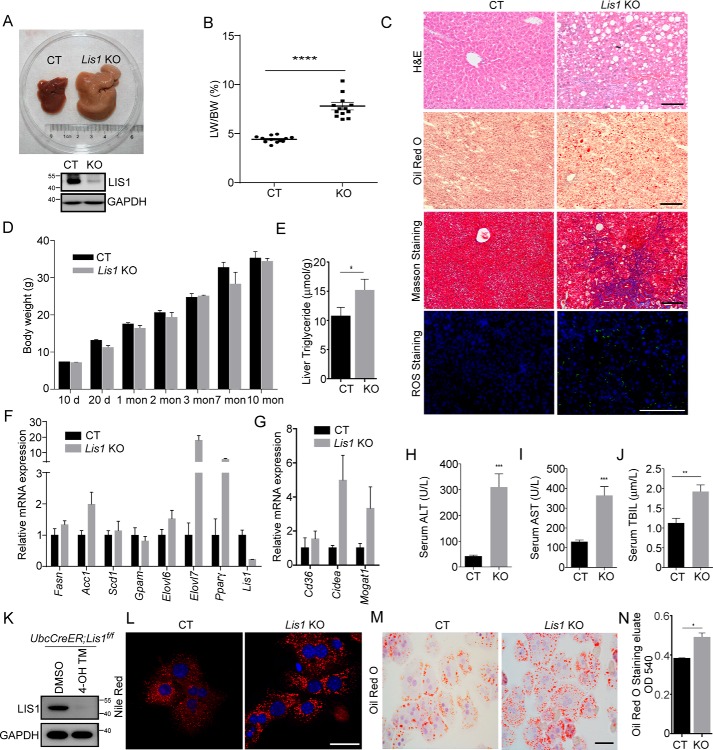
**Liver-specific knockout of *Lis1* results in fatty liver in mice.** *A,* gross morphology of control (*left*) and *Lis1* KO (*right*) mouse liver at 2 months. The *Lis1* mutant displayed an enlarged liver. *B,* the ratio of liver weight (*LW*) to gross body weight (*BW*) of control and *Lis1* KO mice at 2 months. *C,* stained sections of livers from control and *Lis1* KO mice at 2 months with the indicated methods. Increased lipids, fibrosis, and ROS were observed in *Lis1* KO livers. *D,* body weight of control and *Lis1* KO mice at different ages. *E,* liver triglyceride quantification; *n* = 5. *F,* gene expression analysis by quantitative PCR showing increased lipogenesis in *Lis1* KO livers; *n* = 4. *G,* gene expression analysis by quantitative PCR showing increased lipid uptake and storage in *Lis1* KO livers; *n* = 4. *H–J,* quantifications of serum ALT (*H*), AST (*I*), and TBIL (*J*). Severe liver injury was observed in *Lis1* KO mice. *K,* Western blotting analysis of *UbcCreER;Lis1^f/f^* hepatocytes treated with DMSO or 4-OH tamoxifen (*4-OH TM*) with the indicated antibodies. *L,* Nile Red staining of liver sections from control and *Lis1* KO primary hepatocytes. Accumulation of lipids was found in primary hepatocytes in the absence of *Lis1. M,* Oil Red O staining of liver sections from control and *Lis1* KO primary hepatocytes. *N,* relative amount of Oil Red O eluted from control and *Lis1* KO primary hepatocytes was measured by spectrophotometer; *n* = 3 per group. The *scale bars* represent 100 μm in *C*, 10 μm in *L*, and 20 μm in *M*.

Global deletion of *Lis1* caused early embryonic lethality (12). Therefore, to understand the physiological role of *Lis1* in adult liver homeostasis, we specifically deleted *Lis1* in the hepatocytes using *AlbCre* transgenic mice, in which the Cre recombinase is under the control of the liver-specific albumin promoter and a “floxed” allele of *Lis1*. Expression of Cre results in roughly 40% recombination in hepatocytes at birth and almost complete recombination by 2 weeks after birth (29). *AlbCre;Lis1^f/f^* (referred as *Lis1* KO) mice were born at the expected Mendelian ratio. *Lis1* KO mice were fertile, and displayed no apparent abnormality. To examine the efficiency of *Lis1* deletion in the liver, we isolated livers from 6–8-week-old mutant mice. Western blot analysis confirmed that LIS1 protein levels were greatly reduced in *Lis1* KO livers (Fig. 1*A*). Closer examination of LIS1 proteins by immunohistochemistry showed that >95% hepatocytes lost the expression of Lis1 (Fig. S1). These results also indicate that there is no compensatory proliferation of *Lis1*-expressing hepatocytes to regenerate *Lis1*-deficient liver. Livers of *Lis1* KO mice appeared paler and significantly larger than the controls with 100% penetrance (Fig. 1*A*). The ratio of liver weight to body weight increased from roughly 5% in the control mice to 8% in *Lis1* KO mice (Fig. 1*B*). Hematoxylin and eosin (H&E) staining showed that *Lis1* KO hepatocytes were markedly vacuolated (Fig. 1*C*). When liver sections from these mutant mice were stained for neutral lipids using Oil Red O staining, there was a significant increase in the amount of lipids in *Lis1* KO mice compared with the controls, demonstrating a defect in lipid metabolism (Fig. 1*C*). Further analysis revealed that hepatic steatosis was induced by loss of *Lis1*, as measured by quantification of the triglyceride (TG) extracted from livers by a colorimetric assay (Fig. 1*E*).

Next, we investigated the lipid metabolic pathways in *Lis1* KO livers. The expression of genes for lipogenesis, lipid uptake, and storage was increased in *Lis1* KO livers compared with the controls (Fig. 1, *F* and *G*), suggesting that increased lipogenesis may be a contributing factor for hepatic steatosis. Notably, although fatty liver disease is often associated with obesity (3, 30), the body weight of *Lis1* KO mice was comparable with control mice at different ages (Fig. 1*D*). Thus, *Lis1* deficiency results in fatty liver without obesity.

Hepatic steatosis is often self-limited, but it can progress to NASH. NASH is distinguished from simple steatosis by the presence of hepatocyte injury (hepatocyte ballooning and cell death), an inflammatory infiltrate, and/or collagen deposition (fibrosis). Next we examined whether *Lis1* KO mice developed liver fibrosis. Indeed, areas of liver fibrosis were frequently found in *Lis1* KO livers as indicated by Masson's staining (Fig. 1*C*). Greatly increased reactive oxygen species (ROS) was often observed in *Lis1* KO livers (Fig. 1*C*). We also observed elevated levels of serum ALT (alanine transaminase), AST (aspartate aminotransferase), and TBIL (total bilirubin) in *Lis1* KO mice (Fig. 1, *H–J*), indicating that severe liver injury appeared in *Lis1* KO mice.

To delineate the events that occur after acute loss of *Lis1* in hepatocytes, we used a tamoxifen-inducible Cre (UbcCreER) (31), which switches *Lis1* flox allele to null allele after administration of tamoxifen to the culture medium. Mouse primary hepatocytes were isolated from 2-month-old *UbcCreER;Lis1^f/f^* mice. The expression of LIS1 in DMSO-treated (referred as CT) and 4-OH tamoxifen-treated hepatocytes (referred as *Lis1* KO) was verified with Western blotting (Fig. 1*K*). As shown in Fig. 1*L*, *Lis1* deletion led to a significant increase in the number and size of lipid droplets in primary mouse hepatocytes by Nile Red staining. A similar study was performed using Oil Red O for quantification of lipid contents, and the same results were obtained (Fig. 1, *M* and *N*). Thus, acute loss of *Lis1* also results in the accumulation of lipids in primary hepatocytes.

### Analysis of TG secretion and insulin signaling in Lis1 KO livers

**Figure 2.**
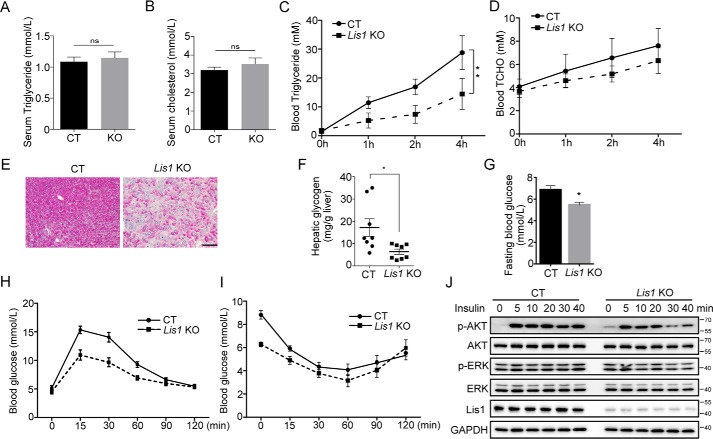
**Triglyceride secretion and glucose homeostasis are disturbed in *Lis1* KO mouse livers.** *A* and *B,* quantification of serum triglyceride (*A*) and cholesterol (*B*) in 2-month-old control and *Lis1* KO mice; *n* = 5. *C* and *D,* quantification of serum triglyceride (*C*) and cholesterol (*D*) in a VLDL-TG secretion assay performed in 2-month-old control and *Lis1* KO mice; *n* = 5. VLDL-TG secretion was affected in *Lis1* KO mice. *E,* PAS staining on the sections from control and *Lis1* KO livers. Decrease in glycogen was detected by PAS staining. *F,* quantification of hepatic glycogen. *Lis1* KO mice had reduced glycogen in the liver. *G,* quantification of fasting blood glucose levels; *n* = 5. *H* and *I,* the glucose tolerance test (*H*) and insulin tolerance test (*I*) were performed in 2-month-old control and *Lis1* KO mice; *n* = 5. *J,* Western blot analysis of control and *Lis1* KO primary hepatocytes treated with insulin for the indicated times. Phosphorylation of AKT was attenuated in *Lis1* KO primary hepatocytes in response to insulin. The *scale bar* represents 100 μm in *E*.

Despite increased lipid content in the livers of *Lis1* KO mice (Fig. 1, *C* and *E*), the levels of plasma TG and cholesterol in *Lis1* KO mice were similar to the controls (Fig. 2, *A* and *B*), suggesting that *Lis1* KO mice may have reduced very-low-density lipoprotein (VLDL)–TG secretion, the lipoprotein responsible for hepatic lipid export. In a well-established VLDL–TG secretion assay, *Lis1* KO mice had a 50% reduction in VLDL–TG secretion (Fig. 2*C*) but not cholesterol (Fig. 2*D*). Thus, lack of *Lis1* in liver affects VLDL–TG secretion, which contributes to the accumulation of lipids in hepatocytes.

Next, we investigated glucose metabolism in *Lis1* KO mice. periodic acid-Schiff (PAS) staining revealed a significant reduction of glycogen in *Lis1* KO livers (Fig. 2, *E* and *F*). To investigate systemic insulin sensitivity, we performed glucose tolerance tests (GTT) and insulin tolerance tests in *Lis1* KO mice and controls. We found that despite the marked accumulation of triglyceride in the livers, *Lis1* KO mice had lower fasting blood glucose concentrations compared with the control mice (Fig. 2*G*). In GTT and insulin tolerance test assays, the kinetics of glucose concentrations in *Lis1* KO animals had similar fashion as the controls, with a subtly lower peak and bottom concentrations (Fig. 2, *H* and *I*). Thus, despite hypoglycemia in *Lis1* KO mice, these animals were more tolerant to glucose and insulin than the controls. We also found that the activation of insulin-dependent phosphorylation of AKT was attenuated as a result of *Lis1* depletion in isolated primary hepatocytes in response to insulin (Fig. 2*J*). Thus, these data indicate that *Lis1* KO mice had reduced VLDL–TG secretion and aberrant glucose metabolism, which also contribute to the development of fatty liver.

### RNA-Seq analysis reveals that deletion of Lis1 causes hepatic inflammation

**Figure 3.**
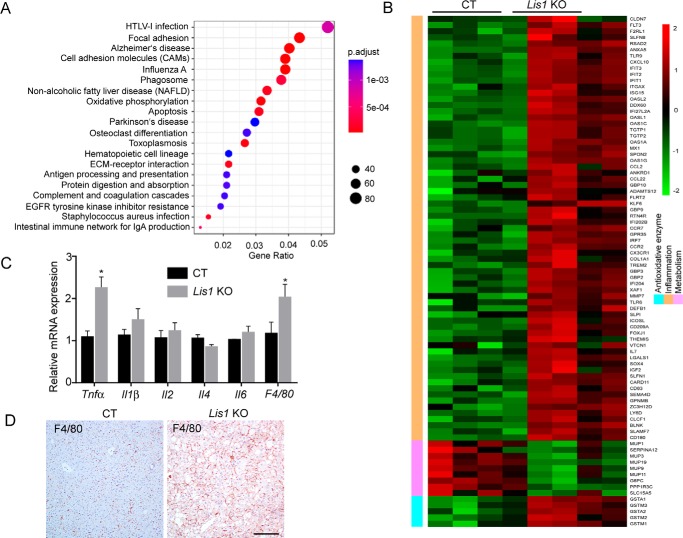
**RNA-Seq analysis of *Lis1* KO livers.** *A,* top pathways that were enriched in differentially expressed genes identified by RNA-Seq analysis in *Lis1* KO livers compared with control livers. *B,* heat map analysis of the highly up-regulated (*red*) and down-regulated (*green*) metabolism, inflammatory and antioxidant genes in *Lis1* KO livers compared with controls. *C,* quantitative PCR analysis of cytokines in control and *Lis1* KO livers; *n* = 4. *D,* immunohistochemistry staining revealed increased macrophages in *Lis1* KO livers by F4/80 antibody; *n* = 5.

To further uncover the molecular mechanisms that underlie the pathogenesis of *Lis1*-deficient liver, we performed RNA-Seq analysis by using RNA isolated from *Lis1* KO and control mice livers (Fig. S2*A*). Among the differentially expressed genes, 1257 were up-regulated and 252 were down-regulated (Fig. S2*A*, Table S1). Ingenuity Pathway Analysis of RNA-Seq data identified a number of pathways that were enriched in the differentially expressed genes (Fig. 3*A*), such as focal adhesion, cell adhesion molecules, apoptosis, protein digestion, and absorption etc. Importantly, NAFLD was among the most significantly affected pathways, which further supports our phenotypic analysis. Furthermore, compared with the controls, the *Lis1* KO mice had increased expression of a battery of inflammation-related genes (Fig. 3*B*). Analysis of a public human fatty liver data set (GSE48452) also revealed significantly increased inflammation-related genes (Fig. S2*B*). Changes in cell adhesion molecules were both found in *Lis1* KO livers and GSE48452 data set (Fig. 3*A* and Fig. S2*B*). Infiltration of macrophages into tissues is a hallmark of local inflammation. Analysis of mRNA from livers of *Lis1* KO mice revealed two times increase in macrophage markers indicated by the expression of macrophage inflammation protein F4/80 (Fig. 3*C*). F4/80 immunostaining further confirmed this finding (Fig. 3*D*). Notably, TNFα and IL-1β, two major proinflammatory cytokines were also significantly increased in the livers of *Lis1* KO mice (Fig. 3*C*). These observations indicate that *Lis1* KO mice are prone to develop hepatic inflammation.

### Deletion of Lis1 results in elevated ER stress in the liver

**Figure 4.**
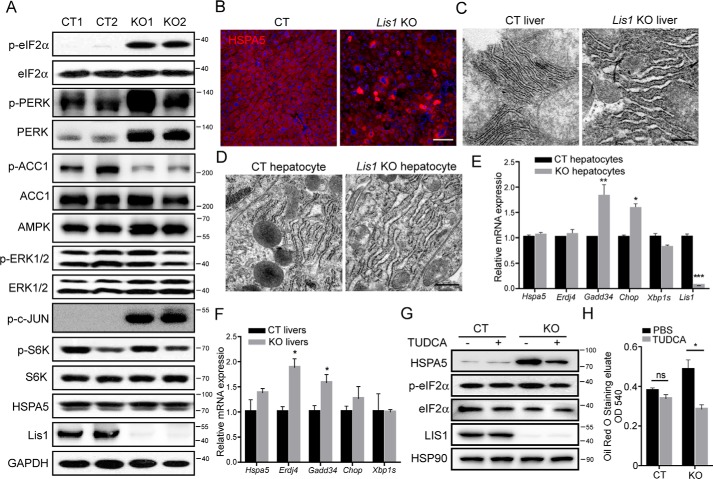
**Loss of *Lis1* in mouse livers results in an elevated ER stress response in association with an enlarged ER size.** *A,* Western blot analysis of control and *Lis1* KO mouse livers at 2 months with the indicated antibodies. *B,* immunofluorescent analysis of livers from control and *Lis1* KO mice with HSPA5 antibody. HSPA5 expression was substantially increased in 10% *Lis1* KO hepatocytes. *C,* representative TEMs of 2-month-old control and *Lis1* KO mouse livers. Enlarged ER was found in *Lis1* KO hepatocytes. *D,* representative TEMs of control and *Lis1* KO primary hepatocytes. *E,* quantitative PCR analysis in control and *Lis1* KO primary hepatocytes; *n* = 4. *F,* quantitative PCR analysis in control and *Lis1* KO livers at 3 months; *n* = 3. *G,* Western blot analysis of control and *Lis1* KO primary hepatocytes with the indicated antibodies using the indicated treatment. p-eIF2α and HSPA5 expression was elevated after acute deletion of *Lis1 in vitro*. TUDCA treatment reduced the expression of p-eIF2α and HSPA5 in *Lis1* KO hepatocytes. *H,* quantification of relative Oil Red O amount in primary control and *Lis1* KO hepatocytes treated with DMSO or TUDCA; *n* = 3. The *scale bars* represent 100 μm in *B*, 500 nm in *C* and *F*.

Activation of ER stress induces inflammation (32), perturbs hepatic lipid metabolism (33), and results in the development of fatty liver. We therefore hypothesized that *Lis1* deletion might induce ER stress. ER stress provokes an ER transmembrane protein kinase, pancreatic ER kinase (PERK), to phosphorylate a subunit of the translation initiation factor (eIF2α) thus blocking cellular protein translation (34, 35). We examined whether ER stress underlies the development of fatty liver in *Lis1*-deficient mice. As shown in Fig. 4*A*, *Lis1* KO livers displayed increased ER stress, as evidenced by the induction of eIF2α phosphorylation. Interestingly, PERK expression was also greatly increased (Fig. 4*A*). Hyperactivation of JNK (c-Jun N-terminal kinase) is another marker of ER stress (36). The phosphorylation of c-Jun, JNK downstream target, was significantly increased in *Lis1* KO mice compared with control mice (Fig. 4*A*). AMPK (AMP-activated protein kinase) has been suggested to be a central player regulating fatty acid metabolism through its ability to suppress ACC (acetyl-CoA carboxylase) activity by phosphorylating it, and considered as a potential drug target for treating NAFLD (37). Importantly, the phosphorylation of ACC was significantly reduced in *Lis1* KO livers (Fig. 4*A*), indicating a decrease in AMPK activity. Phosphorylation of S6K (ribosomal S6 kinase), an mechanistic target of rapamycin substrate, was normal in *Lis1* KO mice compared with the control mice (Fig. 4*A*). Although HSPA5 (heat shock protein family A (Hsp70) member 5, BIP, GRP78) protein levels, one of ER stress response genes, were unchanged in Western blot assay (Fig. 4*A*), immunohistochemistry staining showed increased HSPA5 expression in about 10% hepatocytes of *Lis1* KO livers (Fig. 4*B*). In addition, TEMs (transmission electron micrographs) showed the remarkable enlargement of the ER compartments in the *Lis1* KO livers (Fig. 4*C*) and *Lis1* KO primary hepatocytes (Fig. 4*D*). Taken together, these data suggest that there is elevated ER stress in *Lis1* KO livers.

Similar results of increased ER stress were found in the primary hepatocytes when *Lis1* was acute and removed by administration of 4-OH tamoxfien. Deletion of *Lis1* induced the expression of ER stress response genes, such as *Chop* and *Gadd34* in primary hepatocytes and mouse livers (Fig. 4, *E* and *F*), and eIF2α phosphorylation and HSPA5 expression in primary hepatocytes (Fig. 4*G*). TUDCA (tauroursodeoxycholic acid), an endogenous bile acid, is a well-known ER chaperone that alleviates ER stress (30). Importantly, HSPA5 expression and lipid content were markedly decreased when *Lis1*-deleted hepatocytes were treated with TUDCA (Fig. 4, *G* and *H*), suggesting that ER stress causes the increased lipid contents in *Lis1* KO hepatocytes. Together, these findings indicate that deletion of *Lis1* in mouse liver results in elevated ER stress and hepatic steatosis. TUDCA could attenuate lipid accumulation in *Lis1* deficient hepatocytes.

### Hepatocytes from Lis1 KO mice and human fatty livers display diffused Golgi stacks

Inactivation of LIS1 or its interacting protein NDEL1 results in the fragmentation of Golgi and the defect of vesicle transport. In the secretory pathway, proteins are transported from the ER to Golgi intermediate compartment (ERGIC) to trans-Golgi cisternae and packed in secretory vesicles for fusion with the plasma membrane. The defect in ERGIC or Golgi may affect the secretion of TG and leads to the accumulation of lipids in hepatocytes.

**Figure 5.**
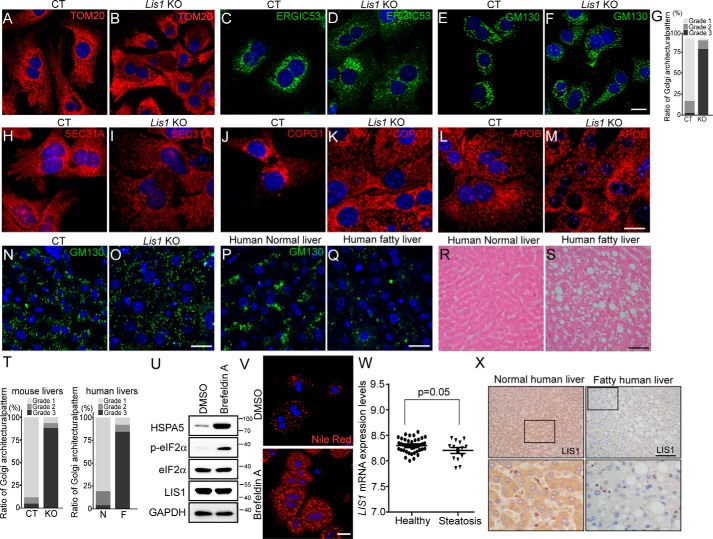
**Hepatocytes in *Lis1* KO mouse and human fatty livers display diffused Golgi stacks.** *A–F,* immunofluorescent staining of control and *Lis1* KO primary hepatocytes with the indicated antibodies revealed the disorganization of mitochondria and Golgi stacks. *G,* quantification of the Golgi architectural changes in *E* and *F. H–K,* immunofluorescent staining with the indicated antibodies revealed the disorganization of COPII (*H* and *I*) and COPI (*J* and *K*) vesicles in *Lis1* KO primary hepatocytes. *L* and *M,* immunofluorescent staining with APOB antibody revealed the abnormal distribution of APOB vesicles in *Lis1* KO primary hepatocytes. *N* and *O,* immunofluorescent staining of control and *Lis1* KO mouse livers with GM130 antibody. *P* and *Q,* immunofluorescent staining of human normal liver and fatty liver sections with GM130 antibody revealed disruption of normal Golgi structure in human fatty livers *in vivo. R* and *S,* H&E staining of human normal liver and fatty liver sections. *T,* quantifications of the architectural changes of the Golgi apparatus by GM130 staining in *N–Q. N*, normal human liver; *F*, fatty human liver. *U,* Western blotting analysis revealed elevated p-eIF2α expression in brefeldin A-treated primary hepatocytes. *V,* staining of primary hepatocytes using Nile Red revealed the accumulation of lipids in brefeldin A-treated cells. *W,* relative *LIS1* mRNA levels in the livers of human healthy controls and NAFLD samples from data set GSE48452. *X,* immunohistochemistry staining of human normal and fatty liver sections with LIS1 antibody. LIS1 expression was reduced in human fatty livers. Higher magnification of *boxed areas* is shown in the *lower panel*. The *scale bars* represent 10 μm in *A–F*; 20 μm in *H–M*; 50 μm in *N–Q*; and 25 μm in *V*; and 100 μm in *R*, *S*, and *X*.

Consistent with previous study, disruption of LIS1 led to mitochondria fragmentation as shown by TOM20 staining (Fig. 5, *A* and *B*). The possible involvement of LIS1 in ER-Golgi transport was subsequently evaluated by immunofluorescence staining of the ERGIC marker protein ERGIC53, and the Golgi marker protein GM130. As shown in Fig. 5, *C–G*, Golgi and ERGIC were localized around the nucleus in control primary hepatocytes, however, Golgi stacks and ERGIC were fragmented and distributed in the entire cytoplasm in *Lis1* KO primary hepatocytes. Coat protein complex I (COPI) and COPII vesicles are essential trafficking machineries of the conventional protein secretory pathway cycling between the ER and Golgi. COPI vesicles mediate transport of proteins and lipids from the Golgi to the ER, whereas COPII mediates transport of cargoes from the ER to the Golgi (38). In *Lis1* KO hepatocytes, COPII vesicles marked by SEC31A were less than in the controls and their distribution was more disperse in the cytoplasm (Fig. 5, *H* and *I*). COPI vesicles were accumulated around nuclei in the control hepatocytes, but distributed in the entire cytoplasm with large vesicles in *Lis1* KO hepatocytes (Fig. 5, *J* and *K*). Similar results were observed in LIS1 knockdown HeLa cells (Fig. S3). Furthermore, we examined the localization of APOB, the major structural proteins in VLDL. In control hepatocytes, APOB containing vesicles formed tubular networks (Fig. 5*L*). However, they were fragmented in *Lis1* KO hepatocytes and formed larger vesicles (Fig. 5*M*). These results suggest that loss of *Lis1* alters the distribution of membrane containing vesicles and may affect the secretion of VLDL in hepatocytes.

Next, we asked whether Golgi architecture was disrupted in the livers of *Lis1* KO mice. As expected, Golgi stacks were dispersed in the cytoplasm of hepatocytes in *Lis1* KO mouse livers (Fig. 5, *N*, *O*, and *T*). Next, we examined the distribution of the Golgi apparatus in human fatty liver and normal human liver samples. Interestingly, Golgi stained by GM130 was distributed in a scattered pattern in 70% (7 of 10) of human fatty livers instead of a compact stack associated with THE nucleus in normal human livers (Fig. 5, *P*, *Q*, and *T*). H&E staining revealed that all of the human fatty liver samples displayed characteristic hepatic steatosis phenotype (Fig. 5, *R* and *S*). These findings suggest that the aberrant distribution of Golgi and ERGIC may involve the progression of hepatic steatosis.

To test whether disruption of ER-Golgi transport could cause accumulation of lipid in hepatocytes, we used brefeldin A, an ER-Golgi transport inhibitor, which has been shown to cause the collapse of the Golgi, accumulation of proteins in the ER, and ER stress (39). Consistent with that study, the collapse of the Golgi triggered activation of ER stress and resulted in lipid accumulation in primary hepatocytes (Fig. 5, *U* and *V*).

Moreover, we analyzed a public data set GSE48452 and found a correlation between *LIS1* expression and human hepatic steatosis (Fig. 5*W*). Furthermore, immunostaining of human fatty liver samples with LIS1 antibody revealed the down-regulation of LIS1 expression (6/10) (Fig. 5*X*), suggesting reduced LIS1 expression may involve in the pathogenesis of fatty liver in human.

### Lack of Lis1 increases genomic instability and tumorigenesis in the liver

**Figure 6.**
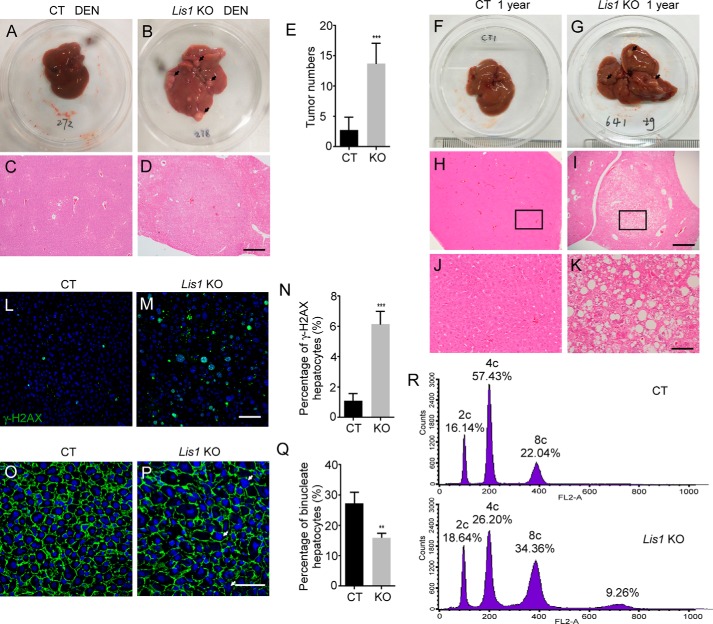
**Lis1 regulates hepatocyte ploidy, genomic instability, and tumorigenesis in the liver.** *A* and *B,* morphology of control and *Lis1* KO mouse livers 6 months after DEN injection. *Arrows* indicate tumors. *C* and *D,* H&E staining of control and *Lis1* KO mouse livers treated with DEN. *E,* quantification of tumor numbers from control and *Lis1* KO mouse livers treated with DEN; *n* = 5. *F* and *G,* morphology of 1-year-old control and *Lis1* KO mouse livers. *Arrows* indicate premalignant nodules. *H* and *I,* H&E staining of 1-year-old control and *Lis1* KO mouse livers. *J* and *K,* higher magnification of H&E staining from the *boxed areas* in *H* and *I,* respectively. *L* and *M,* immunofluorescent staining of 2-month-old control and *Lis1* KO livers with γH2AX antibody. Increased γH2AX positive hepatocytes were observed in *Lis1* KO livers. *N*, quantification of γH2AX-positive hepatocytes in 2-month-old control and *Lis1* KO livers; *n* = 5. *O* and *P,* immunofluorescent staining of livers with β-catenin antibody and DAPI from 2-month-old control and *Lis1* KO mice. β-Catenin demarcates cell boundaries. *Arrows* indicate large nuclear in *Lis1* KO hepatocytes. *Q,* quantification of the ratio of binucleate hepatocytes in 2-month-old control and *Lis1* KO mice; *n* = 5. *R,* FACS analysis of the hepatocyte ploidy from 2-month-old control and *Lis1* KO livers. Increased hepatocyte polyploidy was observed in *Lis1* KO hepatocytes. The *scale bars* represent 500 μm in *C*, *D*, *H,* and *I;* 100 μm in *J* and *K;* 50 μm in *L* and *M;* and 25 μm in *O* and *P*.

Our previous study showed that LIS1 expression was reduced in human liver cancer samples (28). Then, we investigated whether loss of *Lis1* increases the susceptibility to liver tumor development. We induced liver tumors in control and *Lis1* KO mice by injecting diethylnitrosamine (DEN) at postnatal day 14 and examined the development of liver tumors 6 months later. Both groups developed visible liver tumors (Fig. 6, *A* and *B*). However, *Lis1* KO mutant mice developed a greater number and a much larger volume of liver tumors than controls (Fig. 6*E*). Liver histology revealed the hepatic lesions in *Lis1* KO mutants (Fig. 6, *C* and *D*). Our data suggest that *Lis1* KO mice are more susceptible to carcinogen-induced liver tumor formation.

Next, we examined the livers obtained from 1-year-old *Lis1* KO mice and found that these mice showed frequent premalignant nodules featuring clear-cell dysplasia by histological analysis (Fig. 6, *F–K*, *n* = 10/10). Immunostaining for phosphorylated histone H2AX (γH2AX) demonstrated a substantially higher chromosomal instability in the 2-month-old *Lis1* KO mutants (Fig. 6, *L–N*), indicating increased DNA damage in *Lis1* KO livers. Thus, loss of *Lis1* increases genomic instability and oncogenesis in *Lis1* KO livers.

### Loss of Lis1 leads to increased hepatocyte polyploidy

The vast majority of hepatocytes are polyploidy, either mononucleate or binucleate (40, 41). In mice and humans, polyploidization is believed to occur primarily by failed cytokinesis. Abnormal expression of LIS1 results in mitotic defects. Then we examined whether disturbing *Lis1* function affects hepatocyte mitosis and ploidy. Interestingly, the number of binucleate hepatocytes was significantly decreased in *Lis1* KO livers (Fig. 6, *O–Q*). To characterize the function of *Lis1* in hepatocyte ploidy, we examined the DNA content and nuclear size in the hepatocytes of *Lis1* KO liver tissues. We determined the hepatocyte ploidy by flow cytometry and analysis of DNA contents. We observed that 60% of total hepatocytes in control livers were tetrapoloid (4c), with a population of diploid cells (2c, 16%) and a fraction of octaploid cells (8c, 22%) (Fig. 6*R*). However, in *Lis1* KO mouse livers, only 26% of hepatocytes were 4c and many were 8c (>30%) or greater than 8c (9%) (Fig. 6*R*). Moreover, larger hepatic nuclei were often observed in *Lis1* KO mice (Fig. 6, *M* and *P*), which is consistent with the higher degree of ploidy in *Lis1* KO livers. These results demonstrated that *Lis1* plays a critical role in hepatocyte ploidy regulation.

## Discussion

Heterozygous loss of human LIS1 results in the severe brain malformation disorder. In this study, we uncovered a physiological role of *Lis1* in maintaining metabolic homeostasis and hepatic ploidy. We generated liver-specific *Lis1* KO mice, and found that these mice developed hepatic steatosis, progressive liver injury with fibrosis, and eventually cancer. Our RNA-Seq analysis uncovered a significant number of genes and several pathways were perturbed in *Lis1* KO livers. It is interesting to note that NAFLD was uncovered as a major affected pathway, which further validates our *Lis1* KO liver phenotypes. Although no animal model recapitulates the entire disease spectrum of NAFLD in human (42), *Lis1* KO animal model has revealed several key molecular processes that recapitulate the major features of NAFLD progression: hepatocyte damage, inflammation, fibrosis, and progression to the development of liver tumor.

Hepatocytes are enriched in both smooth and rough ER, where plasma protein, VLDL, and lipoprotein are synthesized and secreted (43, 44). ER stress has been observed in the development of liver diseases, including fatty liver disease in recent years based on studies in human and mouse models. Liver samples from patients with NAFLD and NASH demonstrated increased eIF2α phosphorylation and HSPA5 expression, although other ER stress markers were not increased (45). *Lis1* liver KO phenotype is reminiscent of essential aspects of human fatty liver disease, such as ER stress. ER stress activates eIF2α, JNK, and oxidative stress pathways, which have been observed in *Lis1* KO livers. Interestingly, treatment with TUDCA, which is a chemical chaperone that alleviates ER stress, reduced the lipid accumulation in *Lis1* KO primary hepatocytes. Triglycerides are packed in the liver as VLDLs. Generation of these lipoproteins is initiated in the ER and further maturation occurs in the Golgi (46). In the secretory pathway, membrane vesicles emerge from the ER and deliver nascent secretory or membrane proteins to the cis faces of the Golgi complex for posttranslational modifications such as glycosylation. Modified proteins are transported to trans-Golgi cisternae and packed in secretory vesicles for fusion with the plasma membrane. Instead of passive diffusion, these organelles are actively transported and organized by molecular motors (47, 48). Importantly, the normal architectures of these membrane organelles and the motor function of dynein were disrupted in *Lis1*-deficient hepatocytes, which might lead to the defects in posttranslational modifications and secretions of lipoproteins.

Mitochondria in hepatocytes play a major role in fatty acid oxidation and ATP synthesis. Mitochondrial β-oxidation is one pathway for disposal of fatty acids, but results in generation of ROS. Endogenous antioxidant mechanisms are able to protect against cellular injury from ROS. However, impaired mitochondrial function leads to chronic lipid overload, ROS leads to fatty acid peroxidation that further interferes with mitochondrial function through oxidative damage to mitochondrial DNA and proteins (49). LIS1 is required for proper mitochondrial movement and localization (50). In *Lis1* KO livers, mitochondria are fragmented and there are increased ROS. Several antioxidative enzymes were up-regulated in RNA-Seq analysis in *Lis1* KO livers (Fig. 3*B*). As noted above, those results raise the possibility that there might be impaired mitochondrial function in *Lis1* KO hepatocytes, which contributes to the liver phenotypes of *Lis1* KO mice in conjunction with elevated ER stress.

The role of LIS1 in cancer development is less explored. LIS1 plays an important role in mitosis and precise cell division. Inhibition of LIS1 function leads to defects in congression and segregation of chromosome, the establishment of mitotic spindle pole integrity, maintaining normal centrosome number, and mitotic spindle orientation (10, 13, 21). Two Miller-Dieker syndrome patients with a deletion in the 17p13.3 region, where *LIS1* is located, were reported to develop gallbladder cancer (51) and acute lymphoblastic leukemia (52). However, Zimdahl's (27) studies showed that deletion of *LIS1* in leukemic cell lines and primary human leukemia samples led to reduced colony-forming ability. This discrepancy might be due to the deletion of *LIS1* at different times. In human Miller-Dieker syndrome patients, *LIS1* is deleted before cancer development and the cancer cells derived from these patients may develop strategies to escape the requirement for the function of LIS1 in cell proliferation. In Zimdahl's (27) studies, LIS1 was inactivated in leukemic cancer cells. Our studies found that mice with *Lis1* deletion in the livers are susceptible to develop liver cancer in response to carcinogen. We speculated that cellular transformation is linked to the increased inflammation and genome instability in *Lis1* KO livers. The precise role of LIS1 in cancer initiation and progression needs further investigation.

In summary, we generated a liver-specific *Lis1* knockout mouse model comprising a spectrum of disease from hepatic steatosis, NASH to liver cancer. Our data also suggest that membrane organelle defects caused by *Lis1* inactivation result in ER stress and VLDL–TG secretion defect. Moreover, we found the disorganized Golgi stacks in human NAFDL sample and the correlation between *LIS1* expression and hepatic steatosis in the public data set. Thus, *Lis1* KO mouse model provides a unique opportunity for delineating the molecular pathways that lead to steatosis, NASH, and liver cancer.

## Materials and methods

### Animal husbandry

All animal study protocols were approved by the Zhejiang University Animal Care and Use Committee. *Pafah1b(Lis1)1*tm2Awb/*J* and *Tg(Alb-cre)21Mgn*/*J* mice were obtained from Jackson Laboratory.

### Histology and immunohistochemistry

Mouse livers were dissected and fixed in 4% paraformaldehyde (PFA) overnight at 4 °C. Livers were embedded in paraffin and sectioned at 6 μm. Histology and immunohistochemistry were performed following standard procedures. For Oil Red O staining, liver tissues were fixed in 4% PFA overnight and frozen in OCT compounds. The sections were cut at 10 μm and allowed to air dry. Then the slides were briefly washed with running tap water and rinsed with 60% isopropyl alcohol. The sections were stained with freshly prepared Oil Red O solution (0.3% Oil Red O in 60% isopropyl alcohol) for 15 min and rinsed with 60% isopropyl alcohol followed by lightly staining nuclei with hematoxylin. Human fatty liver samples were purchased from Fanpu Biotech, Inc. (Guilin, China). For ROS staining, mouse livers were fixed in 4% PFA overnight and embedded in OCT compounds, sections were cut at 10 μm. Sections were incubated with dichlorofluorescein diacetate (DCFH-DA) (Beyotime, China) for 30 min at 37 °C. Nuclei were stained with DAPI. For PAS staining, paraffin sections were oxidized in 0.5% periodic acid solution for 5 min, rinsed in distilled water, placed in Schiff reagent for 15 min, then washed in tap water for 5 min and counterstained in Mayer's hematoxylin for 1 min.

### Immunofluorescence staining and Western blotting

Cells were lysed with 1× SDS-PAGE loading buffer (50 mm Tris-Cl, pH 6.8, 2% SDS, 0.1% bromphenol blue, 10% glycerol, 1% β-mercaptoethanol), tissue samples were lysed with RIPA buffer (150 mm NaCl, 50 mm Tris-Cl, pH 7.4, 1% Nonidet P-40, 0.5% sodium deoxycholate, 0.1% SDS, 1 mm PMSF, protease and phosphatase inhibitors) and then added 2× SDS-PAGE loading buffer. Protein samples were resolved on SDS-PAGE and then transferred to PVDF membranes. The membranes were blocked with 5% milk, incubated sequentially with primary and secondary antibodies. Protein expression was determined by ECL detection reagent (Thermo). The following primary antibodies were used: LIS1 (Sigma, number 110K4895), glyceraldehyde-3-phosphate dehydrogenase (Abcam), APOB (Proteintech, 20578-1-AP), GM130 (BD Biosciences, number 610822), phospho-eIF2α-S51 (CST, number 3597), phospho-ACC (CST, number 11818), phospho-P70S6K T421/S424 (CST, number 9204), phospho-P70S6K T389 (CST, number 9234S), P70S6K (CST, number 2708), phospho-ERK1/2 (CST, number 4370), ERK1/2 (CST, number 4695), phospho-AKT S473 (CST, number 4060), pan-AKT (CST, number 4691), eIF2α (HuaAn Biotechnology, number RT1196), HSPA5 (Proteintech, number 11587-1-AP), ERGIC53, (Proteintech, number 13364-1-AP), TOM20 (Santa Cruz, number 11415), phosho-C-Jun-S63 (Abcam, number 32385), SEC31A (Proteintech, 17913-1-AP), COPG1 (Proteintech, 12393-1-AP), phospho-Histone H2A.X S139 (CST, number 9718) PERK (CST, 3192), AMPK (CST, 5831).

### RT-PCR

Total RNA from cells and tissues were extracted with RNAiso Reagent (TaKaRa) following the manufacturer's protocol. cDNA was generated by PrimeScript^TM^ II 1st Strand cDNA Synthesis (TaKaRa). Quantitative PCR was performed using a SYBR Green system (Abmart). The sequences of qPCR primers for mouse genes are listed in Table S2.

### Metabolite measurements

Serum triglyceride, total cholesterol, ALT, and AST were measured using commercial kits (Nanjing Jiancheng Bioengineering Institute, China). For measuring liver triglyceride, livers were homogenized in PBS and lipids were extracted using chloroform and methanol. For measuring glycogen, livers were homogenized in 0.5 n potassium hydroxide. The glycogen was precipitated using methyl alcohol and digested with 0.25 mg/ml of amyloglucosidase (Sigma). We quantified the resultant glucose concentrations using a glucose HK assay (Sigma).

### TEM

Livers were cut in 3 × 1 × 1-mm pieces, and fixed in 2.5% glutaraldehyde at 4 °C overnight. Following washing 3 times with PBS, tissues were post-fixed with 1% osmium tetroxide for 1–2 h, dehydrated through a graded series of ethanol, and embedded in Epon 812. Ultrathin sections were cut, mounted on uncoated copper grids, and stained with 2% uranyl acetate and 1% lead citrate for 12 min each. Pictures were taken using a Hitachi HT7700 electron microscope.

### Treatment of DEN

Twelve-day-old control and *Lis1* KO male mice were injected with DEN (Sigma, 20 mg/kg body weight) by intraperitoneal injection. Mice were sacrificed and livers were dissected 6 months after DEN injection.

### Hepatic triglyceride secretion assay

Mice were injected with 100 μl of 10% tyloxapol (Sigma) by tail vein injection. Blood was collected at 0, 1, 2, and 4 h. Plasma triglyceride was measured in accordance with the manufacturer's instructions (Nanjing Jiancheng Bioengineering Institute).

### Primary hepatocytes culture

We used *UbcCreER;Lis1^f/f^* mice to generate *Lis1* KO primary hepatocytes. Mouse livers were perfused using the two-step collagenase technique. Primary hepatocytes were treated with 4 μg/ml of 4-OH tamoxifen (Sigma) or DMSO for 48 h and then changed to normal medium for 24–48 h. Cells were treated with 0.5 mg/ml of TUDCA (Calbiochem, number 580549) for 18 h.

### Glucose tolerance test and insulin tolerance test

GTT was performed on mice that were fasted for 16 h. Mice were given a single dose (2 g/kg) of d-glucose by intraperitoneal injection. Circulating glucose levels were then measured at the indicated time points after glucose injection. For the insulin tolerance test, mice were fasted for 6 h and intraperitoneally injected with insulin (Beyotime) at 0.6 units/kg body weight. Blood glucose was measured using a glucometer by sampling from the tail at the indicated time points after injection.

### FACS analysis of hepatocyte polidy

Mouse livers were perfused using two-step collagenase technique. The cell suspension was passed through a 40-μm cell strainer. Isolated hepatocytes were frozen in a −80 °C freezer overnight or longer, thawed on ice, resuspended in 500 μl of hypotonic lysis buffer (0.1% Triton X-100, 50 μg/ml of propidium iodide, 100 μg/ml of RNase I, and 0.1% sodium citrate). After incubation on ice for 1 h with occasional gentle shaking, nearly all cells were detached in solution under phase-contrast light microscopy. These cells were then analyzed with FACS Calibur (BD Biosciences). The collected data were analyzed with FlowJo software.

### Quantification of the Golgi architectural changes

The architectural changes of the Golgi apparatus using GM130 immunofluorescent staining was evaluated according to the following modified classification scheme that was originally proposed by Sonoda *et al.* (53): Grade 1, the majority of the Golgi structures were concentrated at the perinuclear region; Grade 2, partially dispersed Golgi structures were loosely concentrated at the perinuclear region; Grade 3, dispersed Golgi structures were located throughout the perinuclear region and the Golgi structures were fragmented and appeared in small punctate patterns throughout the cytoplasm. More than 200 cells in each group were evaluated and scored using this classification scheme to determine the morphological changes in the Golgi apparatus.

### RNA-Seq analysis

Livers from control or *Lis1* KO mice were dissected and RNA was extracted using RNAiso Reagent (TaKaRa). RNA quality was evaluated using the Agilent 2200 Bioanalyzer (Agilent Technologies). Paired-end libraries were prepared using the SureSelect Strand-specific RNA Library Prep kit (Agilent Technologies). Multiplexed sequencing was run in a HiSeq2500 sequencer (Illumina). Read alignment and gene expression levels were analyzed by the Maverix Analytic Platform (Maverix). The data set was culled from differentially expressed genes with a cutoff of at least a 2-fold change in expression levels (*p* value ≤0.05). The pathway enrichment analysis and the network analysis were performed on differentially expressed genes using Ingenuity Pathway Analysis (Ingenuity Systems, Redwood City, CA). The data were deposited in the Gene Expression Omnibus (GEO) database (accession no. GSE108096).

### Statistics

Statistical analyses were performed with a two-tailed, unpaired Student's *t* test. When multiple comparisons were performed, one-way or two-way analysis of variances with Bonferroni-corrected Student's *t* tests as posttests were performed. A *p* value less than 0.05 was considered significant; *, *p* < 0.05; **, *p* < 0.01; ***, *p* < 0.001. Except where otherwise indicated, experiments were repeated three times. Quantitative data were presented as mean ± S.E. from a representative of at least three independent experiments. All images were representative.

## Author contributions

X. L. and L. L. data curation; X. L., A. W., J. L., Q. W., and M. Z. validation; X. L. and H. S. investigation; R. L. and J. J. software; H. S. conceptualization; H. S. resources; H. S. supervision; H. S. funding acquisition; H. S. writing-original draft; H. S. writing-review and editing.

## Supplementary Material

Supporting Information

## Footnotes

2 The abbreviations used are:

NAFLD: nonalcoholic fatty liver disease
NASH: nonalcoholic steatohepatitis
Lis1: Lissencephaly 1
ER: endoplasmic reticulum
ROS: reactive oxygen species
ALT (alanine transaminase): AST (aspartate aminotransferase) and TBIL (total bilirubin
VLDL: very-low-density lipoprotein
TG: triglyceride
PAS: periodic acid-Schiff
GTT: glucose tolerance test
PERK: pancreatic ER kinase
eIF: eukaryotic initiation factor
JNK: c-Jun N-terminal kinase
AMPK: AMP-activated protein kinase
ACC: acetyl-CoA carboxylase
TEM: transmission electron micrograph
TUDCA: tauroursodeoxycholic acid
ERGIC: ER to Golgi intermediate compartment
COP: coat protein complex
DEN: diethylnitrosamine
DAPI: 4′,6-diamidino-2-phenylindole
PFA: paraformaldehyde.
