# Supplementary material for: Hepatic loss of Lissencephaly 1 (Lis1) induces fatty liver and accelerates liver tumorigenesis in mice
Source: J Biol Chem. 2018 Feb 23;293(14):5160–71. doi: 10.1074/jbc.RA117.001474 (PMC5892582; doi:10.1074/jbc.RA117.001474)
Supplement: Supporting Information [file supp_293_14_5160__index.html]

Hepatic loss of Lissencephaly 1 induces fatty liver and accelerates liver tumorigenesis in mice — Role of Lis1 in liver homeostasis — Hepatic loss of Lissencephaly 1 (Lis1) induces fatty liver and accelerates liver tumorigenesis in mice — Role of Lis1 in liver homeostasis — Supporting Information 

# Hepatic loss of *Lissencephaly 1* (*Lis1*) induces fatty liver and accelerates liver tumorigenesis in mice

## Supporting Information

- Figure S1-3 - Figure S1-3
- Supplemental table S1 - Differentially expressed genes
- Supplemental table S2 - primer sequence
