# Supplementary material for: Hepatic loss of Lissencephaly 1 (Lis1) induces fatty liver and accelerates liver tumorigenesis in mice
Source: J Biol Chem. 2018 Feb 23;293(14):5160–71. doi: 10.1074/jbc.RA117.001474 (PMC5892582; doi:10.1074/jbc.RA117.001474)
Supplement: Supporting Information [file supp_RA117.001474_134696_2_supp_78906_p4fcp3.pdf]

Supporting Information

Hepatic loss of *Lis1* induces fatty liver and accelerates liver tumorigenesis in mice

Xiaoling Li, Liansheng Liu, Ailing Wu, Jinqiu Lu, Qingzhe Wu, Mujun Zhao,Hai Song

Supporting Information includes:

Figure S1. Immunohistochemistry staining on control and Lis1 KO liver consecutive sections at 10 months with anti-LIS1 and CK19 antibodies.

Figure S2. Figure S2. Gene expression profile analysis of *Lis1* KO livers and human NAFLD samples.

Figure S3. Localization of COPI and COPII vesicles is altered in *LIS1* knockdown Hela cells.

Table S1. Differentially expressed genes in Lis1 KO livers comparing with controls.

Table S2. The sequences of qPCR primers for mouse genes.

Figure S1 Li *et al*.

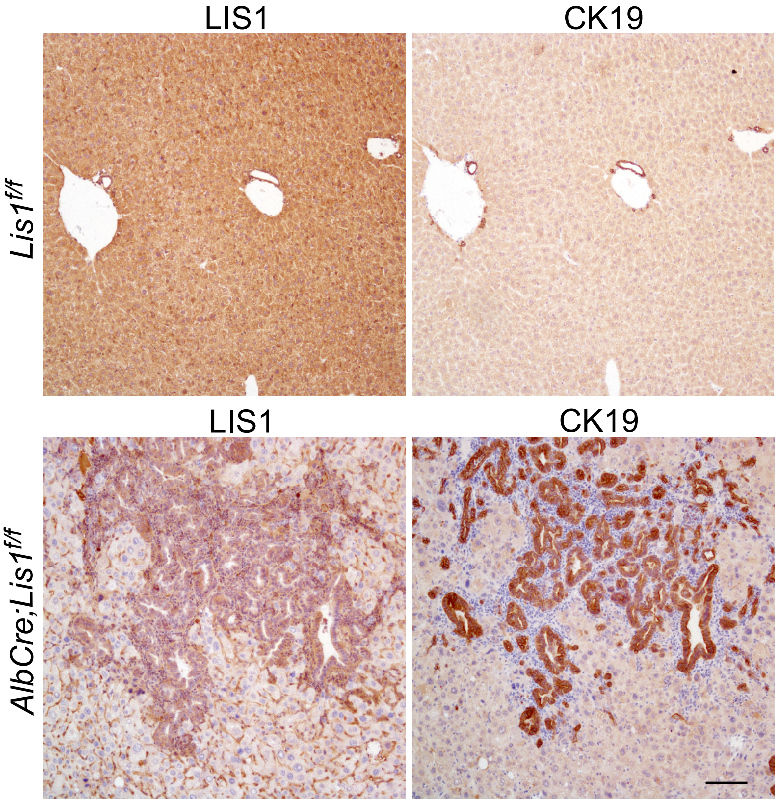

Figure S1. Immunohistochemistry staining on control and Lis1 KO liver consecutive sections at 10 months with anti-LIS1 and CK19 antibodies.
LIS1 is expressed in cholangiocytes and macrophages, but absent in hepatocytes in *Lis1* KO livers, indicating a specific deletion of *Lis1* in hepatocytes using AlbCre driver. Hyperplasia of bile duct was often seen in the older *Lis1* KO mice. The scale bar represents 100 μm.

Figure S2 Li *et al*.

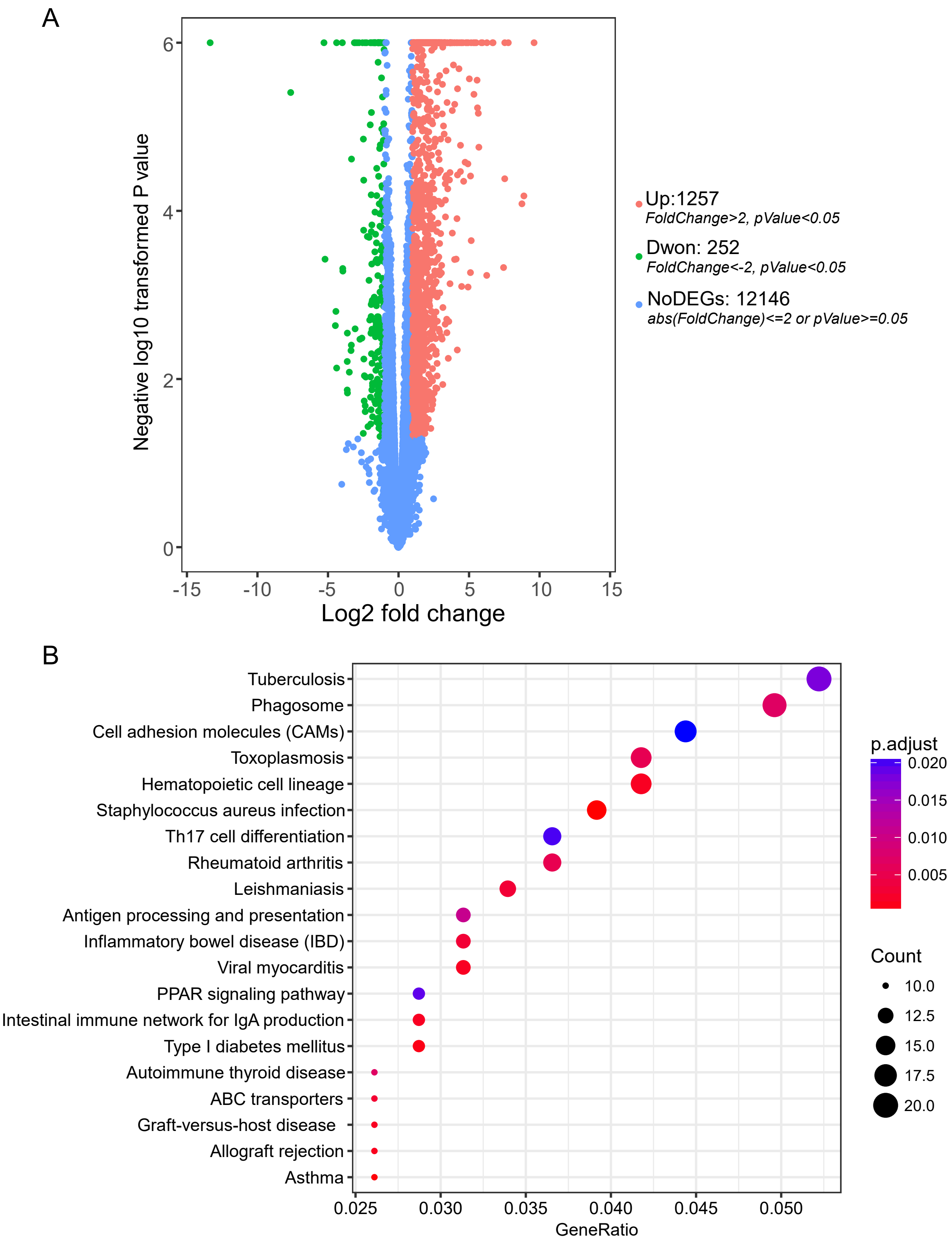

Figure S2. Gene expression profile analysis of *Lis1* KO livers and human NAFLD samples (A) Volcano analysis of gene expression in *Lis1* KO livers using RNA-Seq. (B) Top pathways that are enriched in differentially expressed genes in human NAFLD samples from public data set GSE48452.

Figure S3 Li *et al*.

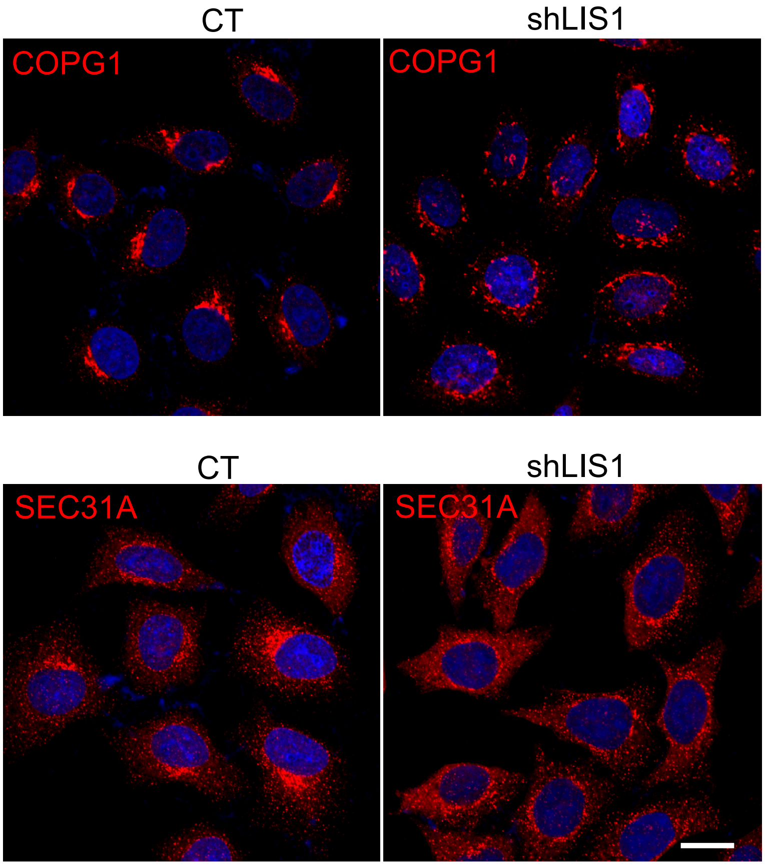

Figure S3. Localization of COPI and COPII vesicles is altered in *LIS1* knockdown Hela cells. Immunofluorescent staining of Hela cells transduced with *shLIS1* lentivirus with anti-COPG1 and SEC31A antibodies. Localization of COPG1 and SEC31A was dispersed in *LIS1* knockdown Hela cells.
