# Supplementary material for: Hepatic loss of Lissencephaly 1 (Lis1) induces fatty liver and accelerates liver tumorigenesis in mice
Source: J Biol Chem. 2018 Feb 23;293(14):5160–71. doi: 10.1074/jbc.RA117.001474 (PMC5892582; doi:10.1074/jbc.RA117.001474)
Supplement: Supporting Information [file supp_RA117.001474_134696_2_supp_78898_p4fcp3.pdf]

Table S2 Li *et al.*

Table S2. The sequences of qPCR primers for mouse genes.

	Forward	Reverse
Hspa5	TGTCTTCTCAGCATCAAGCAAGG	CCAACACTTCCTGGACAGGCTT
Erdj4	AGCCATGAAGTACCACCCTGAC	CGACTATTGGCATCCGAGAGTG
Gadd34	GGCGGCTCAGATTGTTCAAAGC	CCAGACAGCAAGGAAATGGACTG
Chop	GGAGGTCCTGTCCTCAGATGAA	GCTCCTCTGTCAGCCAAGCTAG
Xbp1s	TGGACTCTGACACTGTTGCCTC	TAGACCTCTGGGAGTTCCTCCA
Lis1	GTCTCTGCTTCAGAGGATGCTAC	CTGCTGAACAGGAAGCCAGAAG
Tnfa	GGTGCCTATGTCTCAGCCTCTT	GCCATAGAACTGATGAGAGGGAG
Il1b	TGGACCTTCCAGGATGAGGACA	GTTCATCTCGGAGCCTGTAGTG
Il2	GCGGCATGTTCTGGATTTGACTC	CCACCACAGTTGCTGACTCATC
Il4	ATCATCGGCATTTTGAACGAGGTC	ACCTTGGAAGCCCTACAGACGA
Il6	TACCACTTCACAAGTCGGAGGC	CTGCAAGTGCATCATCGTTGTTC
F4/80	CGTGTTGTTGGTGGCACTGTGA	CCACATCAGTGTTCCAGGAGAC
Fasn	CACAGTGCTCAAAGGACATGCC	CACCAGGTGTAGTGCCTTCCTC
Acc1	GTTCTGTTGGACAACGCCTTCAC	GGAGTCACAGAAGCAGCCCATT
Scd1	GCAAGCTCTACACCTGCCTCTT	CGTGCCTTGTAAGTTCTGTGGC
Gpam	GCAAGCACTGTTACCAGCGATC	TGCAATCAGCCTTCGTCGGAAG
Elovl6	CGGCATCTGATGAACAAGCGAG	GTACAGCATGTAAGCACCAGTTC
Elovl7	TCAGTCGCCAAGAGCAATGAGG	GACATGAAGGAAAGTCACTTGGC
Ppar	GTACTGTCGGTTTCAGAAGTGCC	ATCTCCGCCAACAGCTTCTCCT
Cd36	GGACATTGAGATTCTTTTCCTCTG	GCAAAGGCATTGGCTGGAAGAAC
Cidea	GGTGGACACAGAGGAGTTCTTTC	CGAAGGTGACTCTGGCTATTCC
Mogat1	CCAGCACTACTTTGGCATAATGC	CCTCTAGGTATGTCTGATGCAGC
